# Cortisol, testosterone and psychosocial responses in the assessment of stress in police officers: a brief systematic review of the literature

**DOI:** 10.47626/1679-4435-2023-1153

**Published:** 2024-11-14

**Authors:** Maria Rosário Abrantes, Raquel Barreto Madeira, Luís Fernandes Monteiro, Catarina N. Matias, Luís Miguel Massuça

**Affiliations:** 1 Faculdade de Educação Física e Desporto, Universidade Lusófona, Lisbon, Portugal; 2 Centro de Investigação em Desporto, Educação Física, Exercício e Saúde (CIDEFES) Universidade Lusófona, Lisbon, Portugal; 3 ICPOL - Centro de Investigação, Instituto Superior de Ciências Policiais e Segurança Interna, Lisbon, Portugal

**Keywords:** cortisol, testosterone, saliva, police, stress-disorders, post-traumatic, stress disorders, traumatic, acute, testosterona, saliva, policial, transtornos de estresse pós-traumático, transtornos de estresse traumático

## Abstract

Given the nature of their profession, police officers cannot limit their exposure to stress and trauma, and the endocrine system plays a vital role in regulating and preparing the human body. This study aims to identify studies that have studied the behavior of the hormones cortisol and testosterone in their relationship with the physical and psychological performance of police officers and/or in a training/simulation scenario. The systematic review, limited from 2011 to 2022, was carried out according to the PICO and Preferred Reporting Items for Systematic reviews and Meta-Analyses research strategy, considering seven articles for the critical analysis (classified based on the modified Physiotherapy Evidence Database scale). Of the seven articles considered, (i) five studies are observational, and two are experimental; (ii) 1,475 police officers participated; (iii) three studies evaluated only male participants, and four studies evaluated both sexes; (iv) most studies include salivary collections for hormonal evaluation and questionnaires for behavioral analysis and psychosocial stress; (v) a study analyses salivary collections for hormonal evaluation in response to decision-making tasks; and (vi) a study analyses blood collections for hormonal evaluation. Although studies with proven validity in the association between the hormones cortisol and testosterone and physiological and psychological are scarce, the scientific evidence is consistent and points to these endocrine markers as reliable in quantifying stress levels and performance of police function.

## Abstract (pt)

Dada a natureza da sua profissão, os policiais são incapazes de limitar a sua exposição ao estresse e ao trauma, e o sistema endócrino assume um papel relevante na regulação e preparação do corpo humano. Este trabalho de revisão visa identificar trabalhos que tenham estudado o comportamento dos hormônios cortisol e testosterona na sua relação com o desempenho físico e psicológico de policiais e/ou em cenário de treino/simulação. A revisão sistemática, limitada pelo período entre 2011 e 2022, foi realizada de acordo com a estratégia de pesquisa PICO (acrônimo para P: população/pacientes; I: intervenção; C: comparação/controle; O: desfecho/*outcome*) e a Preferred Reporting Items for Systematic Reviews and Meta-Analyses, sendo considerados sete artigos para a análise integral (classificados com base na escala modificada Physiotherapy Evidence Database). Dos sete artigos considerados, (i) cinco estudos são observacionais e dois são experimentais; (ii) participaram 1.475 policiais; (iii) três estudos avaliaram apenas participantes do sexo masculino, e quatro estudos avaliaram os dois sexos; (iv) a maioria dos estudos compreende coleta de amostras salivares para avaliação dos hormônios e questionários para a análise comportamental e estresse psicossocial; (v) um estudo analisa coleta de amostras salivares para avaliação dos hormônios em resposta a tarefas de tomada de decisão; e (vi) um estudo analisa coleta de amostras de sangue para avaliação dos hormônios. Assim, destaca-se que, apesar de serem escassos os estudos com validade comprovada na associação entre os hormônios cortisol e testosterona e variáveis de caráter fisiológico e psicológico, a evidência científica é consistente e aponta esses marcadores endócrinos como fidedignos na quantificação dos níveis de estresse e desempenho da função policial.

## INTRODUCTION

The study of hormones such as cortisol and testosterone allows us to explore some of the biological processes that link stimuli to behavior.^1^ In fact, the brain is surrounded by chemicals, such as steroid hormones, which can profoundly affect the way the brain works and, in turn, the body’s physical and behavioral responses.^2^

The production of glucocorticoids such as cortisol (stimulated by the adrenal glands) coordinates a series of brain and peripheral systems to mobilize a stress response. Although the stress response involves an adaptation in physiological function that extends far beyond the initial stress experience, brain stress systems regulate the activation of the autonomic nervous system (ANS) and the hypothalamic-pituitary-adrenal (HPA) axis, which can become disturbed in response to traumatic and/or chronic stress.

This is particularly relevant for a population such as police officers, who are often exposed to disturbing and life-threatening trauma when responding to critical incidents. Regardless of the risk to themselves, police officers are involved in threatening situations as part of their duties. Stress can occur in the form of repeated (i.e. chronic) stress or acute traumatic stress in response to a particularly shocking and/or life-threatening event.

The literature shows that chronic exposure to work-related stress and trauma increases occupational health risks, including cardiovascular disease, metabolic syndrome, sleep disorders, post-traumatic stress disorder (PTSD), depression, and other psychiatric disorders.^3-7^ In addition, the effects of increased responsibility inherent in law enforcement are aggravated by exposure to personal threats, i.e.: (i) the occurrence of bodily injury during a robbery showed a direct correlation with symptoms of post-traumatic depression (PTSD);^8^ and (ii) exposure to trauma (with perception of a major threat to physical integrity) is directly associated with symptoms of PTSD (including disassociation, anxiety, and fear).^5^ In light of these factors, HPA axis activity may be deregulated in this population, leading to a high risk of depression,^9^ which may justify the increased risk of suicidal ideas and deaths due to suicide in police officers.^10^

However, the literature highlights that police training, which involves mental preparedness and resilience (centered on regulating physiological arousal and strengthening executive function and perceptual abilities), improves the well-being of police officers and their performance in highly realistic training scenarios.^11,12^ However, (i) police officers generally do not have access to resources and training that could mitigate the effects of stress at work;^13^ and/or (ii) the destructive consequences of stress are not always expected, i.e. some professionals, despite chronic stress or exposure to trauma, have few or no symptoms.^14^

Exposure to stress does trigger some hormonal disorders, namely increased levels of cortisol, an end product of the HPA axis,^15^ and decreased testosterone, an end product of the hypothalamic-pituitary-gonadal (HPG) axis. In other words, (i) the peripheral changes lead to increased alertness, vigilance, and other important functions to deal with threatening and stressful circumstances;^16^ and (ii) behavioral disorders such as aggressiveness, competitiveness, and dominant behaviors.^1^ This is one of the reasons why the concentration of testosterone and cortisol (T:C) is often used as an indicator of the level of stress, and testosterone levels decrease as cortisol levels increase.^16^

This systematic review aims to identify studies that have examined the behavior of cortisol and testosterone as they relate to physical and psychological performance of police officers and/or in training/simulation settings.

## METHODS

This systematic review was conducted according to the PICO search strategy (acronym for P: population/patients; I: intervention; C: comparison/control; O: outcome) and the Preferred Reporting Items for Systematic Reviews and Meta-Analyses (PRISMA).^17^ A systematic search was conducted between November 2021 and January 2022 in five databases (PubMed, SciELO, Google Scholar, ScienceDirect, and b-on), and covered a limited time span between 2011 and 2022. This search allowed to select studies conducted in any country and reported in English or Portuguese, and the following keywords were used: (police OR “police academy Cadets” OR “police trainees” OR “police students” OR “police recruits”) AND (stress OR anxiety OR “coping ability” OR “mental status” OR “social status” OR “acute stress” OR “aptitude testing”) AND (cortisol OR testosterone).

### INCLUSION AND EXCLUSION CRITERIA OF STUDIES

In accordance with the objective defined for this review, the inclusion criteria were: (i) date of publication of the articles, including studies from 2011 to 2021; (ii) journals; (iii) studies with samples of cortisol and/or testosterone in police officers; and (iv) assessment of the psychological state of police officers. The selection process also included the following exclusion criteria: (i) studies with animals or with populations other than police officers; (ii) uninteresting titles and repeated articles; and (iii) the presence of endocrine or psychiatric diseases, and the use of medication or drugs.

**Figure 1:**
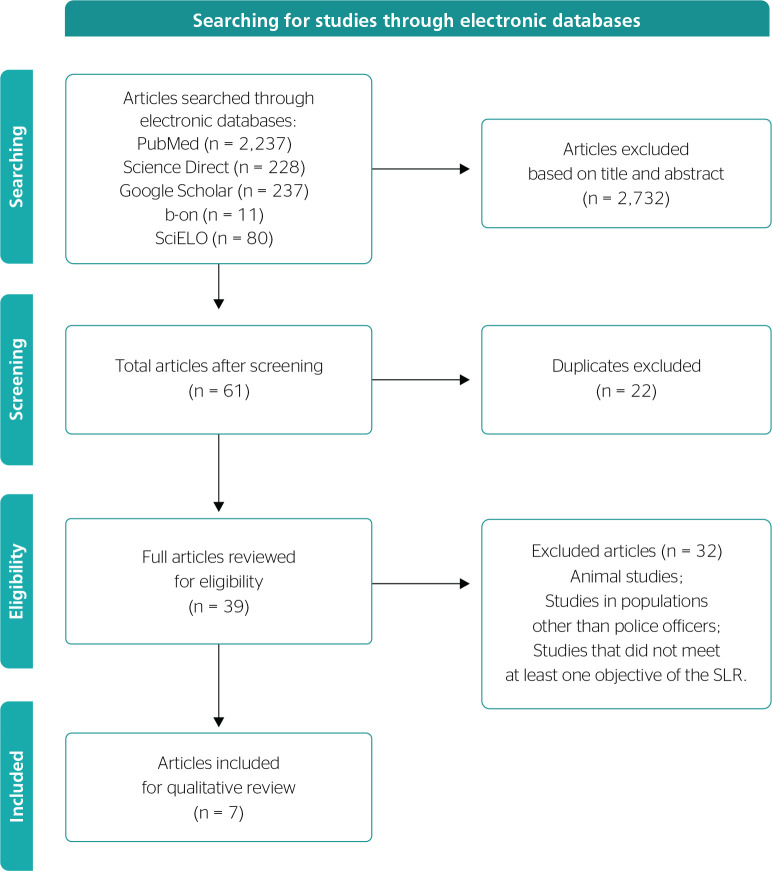
Information flow diagram throughout the different phases of the systematic literature review (SLR).

Potentially relevant studies were selected based on their title, abstract, and full text review (when abstracts did not provide sufficient information for inclusion). The process of article screening and selection is shown below in Figure 1 - information flow diagram based on the PRISMA^17^ recommendations. In addition, Table 1 shows the articles screened to be fully reviewed (n = 7), which were also screened with the modified Physiotherapy Evidence Database scale.^18^

**Table 1:** Characteristics of studies (n = 7) assessing the relationship between cortisol (C) and testosterone (T) and physiological and psychological characteristics in police officers

Authors	Study design	Sample	Evaluation (procedures/instruments)		Main findings	PEDro score
			Hormone	Psychometric or physical		
Dergaa et al.^23^	Cross-sectional	Police officers n = 20; aged 26 ± 2 years	C and T (blood)	RAST	C values increased after the RAST. No significant changes in T levels.	3
Zhang et al.^20^	Cross-sectional	Police officers n = 586 (♀, n = 122; ♂, n = 463); aged 39 years (29-45)	C (saliva)	IPAQ, SCID-I/P, DSM-IV, HAM-D	The mean level of C was higher in police officers with depression.	4
Giessing et al.^19^	Experimental	Police officers (recruits) n = 19 (♀, n = 3; ♂, n = 16); aged 22.84 ± 3.30 years	C (saliva)	Anxiety Thermometer, RSME , SSS-V, SCS, SCS-K-D, vagal HR	The stress scenarios triggered comparable physiological responses (suggesting similar stress levels for both scenarios). Shooting accuracy was low and did not decrease in high stress scenarios. Performance efficiency decreased under stress.	6
Koch et al.^24^	Longitudinal	Police officers n = 340 (♀, 25%; ♂, 75%); control n = 85; aged 18-45	C and T (saliva)	Health questionnaires, NEO-FFI-NL, Cognitive tests, Physical capacity tests	Association: behavioral, psychophysiological, endocrine, and neural alterations of automatic defensive responses. Development of trauma-related symptoms following exposure.	7
Tavares et al.^22^	Cross-sectional	Police officers (military) n = 134; aged 29-34	C (saliva)	ERI scale	Positive association: C at night vs. psychosocial reward. Positive association: C at night vs. effort scores. The GATE explains the ∆C on awakening. GATE, Special Patrol of the Military Police Elite Squad and Motorcyclists explain ∆C, 30 minutes after waking up. GATE and dimension of effort explain ∆C at night.	4
Akinola & Mendes^21^	Experimental	Police officers (patrol) n = 81; aged 40.2 ± 8.33 years	C and T (saliva)	Decision-making task	Police officers with higher cortisol responses to stress made fewer errors in a threat-related decision-making task. There were no associations between testosterone reactivity and shooting errors.	6
Inslicht et al.^25^	Longitudinal	Police officers (recruits) n = 296 (♀, 14%; ♂, 86%); aged 27.4 ± 4.97 years	C (saliva)	Clinical questionnaire SCI for DSM-IV, SCL90-R, GSI, PSQI, LSC-R, CIHQ, ASDS, DSM-IV based, PTSD, PDI, PDEQ	C levels on awakening and accumulated exposure to critical incident stress during training at the academy. It was associated with greater peritraumatic dissociation and greater ASD symptoms during police service assessed at 12, 24, and 36 months. It was not associated with peritraumatic distress or PTSD symptoms.	5

ASD = acute stress syndrome; ASDS = Acute Stress Disorder Scale; C = cortisol; CIHQ = Critical Incident History Questionnaire; DSM-IV = Diagnostic and Statistical Manual of Mental Disorders, 4th edition; ERI = Effort-Reward Imbalance; HR = heart rate; GATE = Grupo de Ações Táticas Especiais; GSI = General Symptom Index; HAM-D = Hamilton Depression Rating Scale; IPAQ = International Physical Activity Questionnaire; LSC-R = Life Stressor Checklist Revised; NEO FFI NL = NEO Five-Factor Inventory Personality Questionnaire; PDEQ = Peritraumatic Dissociative Experiences Questionnaire; PDI = Peritraumatic Distress Inventory; PEDro = Physiotherapy Evidence Database; POMS = Profile of Mood States; PSQI = Pittsburg Sleep Quality Index; PTSD = Post-traumatic stress disorder; RAST = Running-based Anaerobic Sprint Test; RCA = cortisol response on awakening; RSME = Rating Scale Mental Effort; SCL90-R = Symptom Checklist 90-Revised; SCID-I/P = Structured Clinical Interview for DSM-IV-TR Axis I Disorders; SCS = Self Compassion Scale; SCS-K-D = Self Compassion Scale-Short form; SSS-V = Sensation Seeking Scale; T = testosterone; ∆ = delta (variation).

## RESULTS

A total of 2,793 entries were found in the literature search (PubMed, n = 2,237; Google Scholar, n = 237; b-on, n = 11; SciELO, n = 80; ScienceDirect, n = 228). A total of 2,732 studies were excluded based on the title and abstract, and 22 were duplicates. The full texts of the remaining 39 articles were then accessed and reviewed, 32 did not meet the criteria and two were systematic literature reviews. As a result, the search resulted in the inclusion of seven articles evaluating the behavior of police officers and their relationship with psychological and hormonal results (Figure 1).

The studies included were characterized as follows: (i) design - of the seven studies included for review, five were observational (cross-sectional, n = 3; longitudinal, n = 2) and two were experimental; (ii) participants - 1,475 individuals participated in these seven studies; the smallest sample had 19 participants^19^ and the largest had 585 participants.^20^ Of these, three studies evaluated only male participants;^21-23^ four studies compared both sexes;^19,20,24,25^ (iii) instruments and evaluation - most studies (n = 5) included samples of saliva for hormone testing and questionnaires for behavioral and psychosocial stress testing.^19,20,22,24,25^ In addition, one study analyzed samples of saliva for hormone testing in response to decision-making tasks,^21^ and one study^23^ collected blood samples for hormone testing.

As for physiological and hormone responses to physical exercise, Dergaa et al.^23^ observed that short-term peak exercise significantly increased cortisol levels and did not alter testosterone levels.

As for studies centered on the relationship between cortisol, testosterone, and psychological states, it should be noted that: (i) Zhang et al.^20^ found that 15.6% of police officers are depressed (with a higher prevalence in females), cortisol levels in saliva are at their highest on awakening (decreasing in the afternoon), the mean level of cortisol in saliva was higher in police officers with depression, and high levels of cortisol in saliva are associated with an increased prevalence of depression, which may be useful in the early identification of police officers at risk of depression; (ii) Giessing et al.^19^ observed that performance efficiency decreased under stress, and cortisol levels increased following a low-stress scenario and remained high during a high-stress scenario; (iii) Koch et al.^24^ highlighted the correlation between behavioral, psychophysiological and endocrine changes, and neural markers of automatic defensive responses, and also the development of trauma-related symptoms following exposure; Tavares et al.^22^ noted that work overload and demanding responsibilities can cause chronic stress (consequently inhibiting cortisol secretion), suggesting that low cortisol secretion on awakening may be due to metabolic syndrome and/or stress; (iv) Akinola & Mendes^21^ observed that police officers who showed higher cortisol responses to stress made fewer errors in a decision-making task related to threats; and finally, (v) Inslicht et al.^25^ suggest that a higher cortisol awakening response (CAR) represents a pre-exposure risk factor for peritraumatic dissociation and acute stress syndrome (ASD) symptoms during police service.

## DISCUSSION

The studies included in this review have different designs, methods, controls, and data analysis, although they all looked at hormone responses to different challenges. The psychometric evaluation that followed the collection of samples of saliva was presented in the form of various questionnaires.

The results and conclusions of these different studies were grouped into two groups: (i) those that respond to exercise and stress due to load and intensity; and (ii) those that respond to psychological variables and stress due to their performance as police officers.

As for the physiological and hormonal responses to physical exercise, Dergaa et al.^23^ observed increased cortisol levels with short-term peak exercise. This is justified because cortisol plays a role in regulating metabolism during exercise,^26^ since cortisol: (i) helps maintain an adequate supply of glucose in the blood during exercise, increasing the mobilization of amino acids and lipids from skeletal muscle and adipose tissue;^27^ and (ii) facilitates this process by stimulating the liver to create the enzymes involved in the gluconeogenesis and glycogenolysis pathways, which allow the catabolism of glycogen, amino acids, and glycerol into glucose, and by increasing the level of catecholamines, which contribute to greater uptake of carbohydrates.^28^ Dergaa et al.^23^ observed no changes in testosterone levels after short-term peak exercise, contrary to what Smith et al.^29^ found (who reported that high-intensity exercise increased free testosterone and total testosterone in the serum of healthy young men).

As for the relationship between cortisol and psychological states, particularly stress levels and performance of police officers, those who showed a higher cortisol response to stress were found to make fewer errors in decision-making tasks in the face of threats.^24^

A more recent study, however, found that performance efficiency decreased in scenarios with different stress levels, although physiological responses were similar.^19^ In addition, uncertainty, physical threat, and social evaluation have been identified as stress variables in studies investigating the association of performance under stress with endocrine responses (autonomic and emotional), occupational behavior, and stress management capacity.^30^ It highlights that police training should consist of highly realistic scenarios that provide officers with (i) the opportunity to experience how psychological and physiological arousal impacts on their behavior under stress, and (ii) the opportunity to improve performance under extreme stress.

As a matter of fact, police training studies have shown that training with threat-induced anxiety improves perception performance under stress,^31,32^ and therefore the psychoeducation (during training) of police officers should (i) focus on the adaptive function of psychophysiological stress responses, improving work performance in situations of acute stress, and (ii) explain the long-term negative effects of chronic stress responses on physical and mental health.^33^

Kearns et al.^34^ state that most individuals exposed to trauma do not develop PTSD. The current notion is that only individuals at increased risk of PTSD should be targeted for preventive interventions. However, given the high prevalence of PTSD symptoms in police officers, it seems relevant to know the neurobiological mechanisms underlying the development and persistence of trauma-related psychopathology. Nevertheless, more recent studies^24^ report that endocrine imbalances, which occur at both the ascending and descending levels, are directly correlated with PTSD and trauma symptoms, reinforcing associations between behavioral, psychophysiological and endocrine disorders, and neural markers of automatic defensive responses.

Nevertheless, police duties not only entail dangerous and traumatic exposure to events, but also stressful factors linked to poor administrative support, punishment centered on executive philosophies, and high levels of bureaucracy,^35^ and therefore the activity of the HPA axis may be deregulated in this population, potentially increasing the risk of depression.^9^ The relationship between the level of cortisol in saliva and the prevalence of depression in police officers has been widely studied, and it has been observed: (i) that the average level of cortisol in saliva is higher in police officers with depression; (ii) that a high level of cortisol in saliva is associated with a higher prevalence of depression; and (iii) that a high level of cortisol can be useful in identifying police officers at risk of depression.^20^

Besides depression, psychosocial stress and its relationship with cortisol were assessed, and a negative correlation was found between cortisol on awakening and the score obtained in the effort-commitment model used to assess psychosocial stress, while cortisol levels at night were associated (positively) with a reward system.

On the one hand, these considerations can be interpreted in the context of metabolic syndrome^36^ and its associations with health and body composition or, on the other hand, with efficiency and commitment to work, making it difficult to “disconnect” from work at night.^22^ Inslicht et al.^25^ reported an association between CAR and greater peritraumatic dissociation and a higher incidence of ASD symptoms during police duties, prompting the authors to theorize that cortisol on awakening may represent a pre-exposure risk factor for peritraumatic dissociation and ASD symptoms during police duties.

Although cortisol is the classic marker of stress, testosterone has also been used in some studies as an adjunct indicator of stress levels. Akinola & Mendes^21^ reported no association between neuroendocrine reactivity (in this case, testosterone) and shooting errors, regardless of the ethnicity of the subject. Although there were no changes in this hormone levels, testosterone decline is a recognized marker of HPA activation and is associated with energy deprivation. Edwards et al.^37^ observed that testosterone levels are associated with behavioral aspects of social ranking, aggression, dominance, and personal success. Koch et al.,^24^ however, reinforce a correlation between endocrine parameters (testosterone) and symptoms, or at least vulnerability to PTSD and traumatization.

While the literature on the subject in the police population is scarce, it is important to mention that this study has some limitations, namely: (i) the specificity of the population studied (police officers); (ii) the method of saliva collection (not very homogeneous collection times); and (iii) the use of questionnaires (as they are self-assessment measures). This study, however, also seems to reinforce that integrating psychological skills training into police officer basic training curricula can increase positive results, both in situations of acute stress and in long-term health-related consequences.^33^

## CONCLUSIONS

There are seven studies with proven validity on the association between cortisol and testosterone and physiological and psychological variables. The scientific evidence is consistent and points to these endocrine markers as reliable in measuring stress levels and police performance, in addition to their predictive capacity as a marker of PTSD.
